# Management of Spontaneous and Pruritic Keloids: A Case Report

**DOI:** 10.7759/cureus.23227

**Published:** 2022-03-16

**Authors:** Chantae C Hollis, Ariful Alam, Stephanie Pelenyi, Rio Varghese, Nyrie Mann, Chika Kanu-Ivi, Raghu H Nagaraj, Kasi Penumarthi, Maria J Prince, Juaquito Jorge, Frederick Tiesenga

**Affiliations:** 1 Medicine, John F. Kennedy University School of Medicine, Willemstad, CUW; 2 Surgery, West Suburban Medical Center, Oak Park, USA; 3 Medicine, Windsor University School of Medicine, Cayon, KNA; 4 Medicine, Avalon University School of Medicine, Willemstad, CUW; 5 Medicine, Saint James School of Medicine, Park Ridge, USA; 6 Medicine, Caribbean Medical University, Willemstad, CUW; 7 General and Bariatric Surgery, West Suburban Medical Center, Oak Park, USA; 8 General Surgery, West Suburban Medical Center, Oak Park, USA

**Keywords:** keloid management, keloid treatment, spontaneous keloid, keloid scar, keloid

## Abstract

Spontaneous chest wall keloid scars can occur without any history of trauma and are rare. Some keloids present with intense pruritus or paresthesia, prompting patients to seek treatment. Currently, many treatment options are available in medicine. However, for this case report a less invasive treatment modality is evaluated. This clinical case report will present Kenalog-40 injections as a treatment option to treat a spontaneous keloid scar. Observation of this treatment option has illustrated a reduction in size and improvement in pruritus, paraesthesia and discoloration.

## Introduction

Keloids are a type of hyperactive scar formation that appear as thick scars that hyperextend beyond the original wound borders. Keloids are within the spectrum of fibroproliferative disorders that may be mistaken for hypertrophic scars [1]. Both conditions will appear as raised firm cutaneous scars that have abnormal shapes due to excess production of fibrinogen and collagen during the wound healing process [1]. Symptomatically, both may result in pruritus, pain, restricted movement, and are aesthetically displeasing [2]. What sets apart the two is that keloids do not regress and grow beyond the original margins of the initial injury [1]. Keloids typically occur more often in darker pigmented individuals, and there is some evidence that there can be a hereditary tendency [1]. These individuals are also more likely to experience spontaneous keloid formation, which is believed to be caused by microtrauma and minimal cutaneous inflammation [3]. Other risk factors include wound healing by secondary intention [4], repeated trauma, pregnancy, and body piercings [5]. 

There is currently no cure for keloids, and they often recur. First-line treatments consist of an intralesional approach such as triamcinolone acetonide (Kenalog), 5-fluorouracil, verapamil, interferon, bleomycin, and botulinum toxin to be inserted as medication into the papillary dermis [6]. Keloids that persist after an intralesional approach undergo surgical excision as the subsequent step of management [6]. However, it is crucial during the surgical procedure to use corticosteroids as part of polytherapy to prevent recurrence [6]. 

We report a case of a 70-year-old female who presented with a history of a spontaneous and discomforting keloid scar on the anterior chest wall that responded positively to multiple intralesional Kenalog injections.

## Case presentation

A 70-year-old female of Asian descent presented with a 10-year history of a spontaneous, progressively enlarging, and intensely pruritic keloid scar on the anterior mid-chest. The patient reported that the scar was initially less than approximately 1 cm in size but gradually increased over the years and became pruritic about four years ago. The patient attempted treatment with topical 2% over-the-counter cortisone cream without any relief. She recalled receiving an injection of an unknown steroid in the past, which provided minimal relief but caused hyperpigmentation of the scar. The patient presented to her primary care doctor with complaints that the scar had progressively enlarged and became red and itchy with an unbearable "prickling" sensation. She did not recall any trauma to the area, nor any rashes preceding the scar development. The patient did not notice any similar lesions on her body. Her surgical history included a prior cesarean procedure and right unilateral total knee replacement, neither of which developed into keloid scars. 

**Figure 1:**
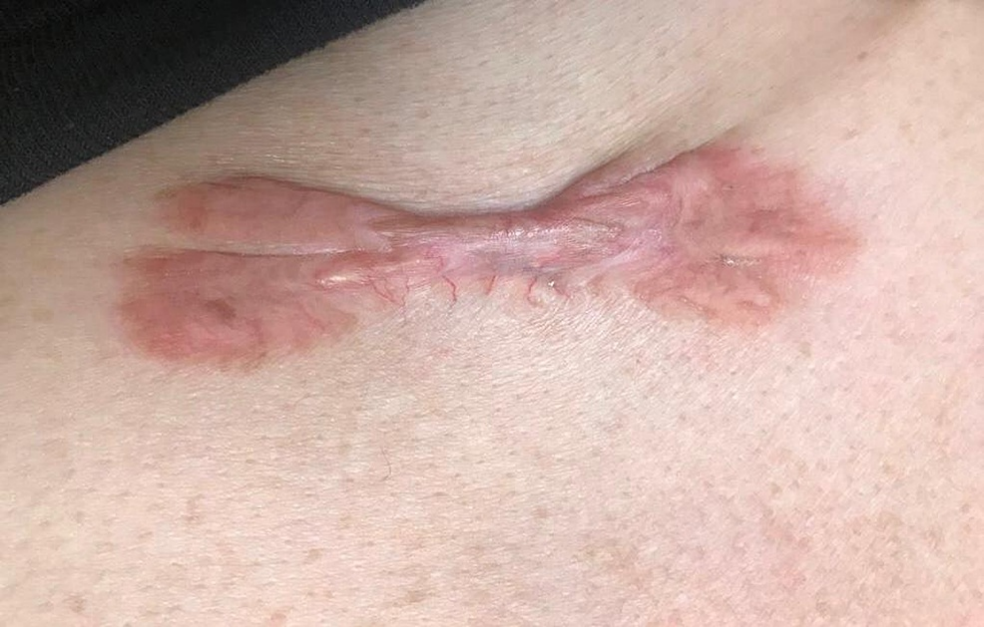
Irregularly shaped keloid on the anterior chest wall

On physical examination, a 7 cm x 4 cm erythematous bow-shaped scar on the anterior mid-chest wall was found (Figure 1). Healed scars on the right knee and abdomen were consistent with knee replacement surgery and a cesarean procedure, respectively. Vital signs showed normal sinus rhythm with elevated systolic and diastolic blood pressures. 

**Figure 2:**
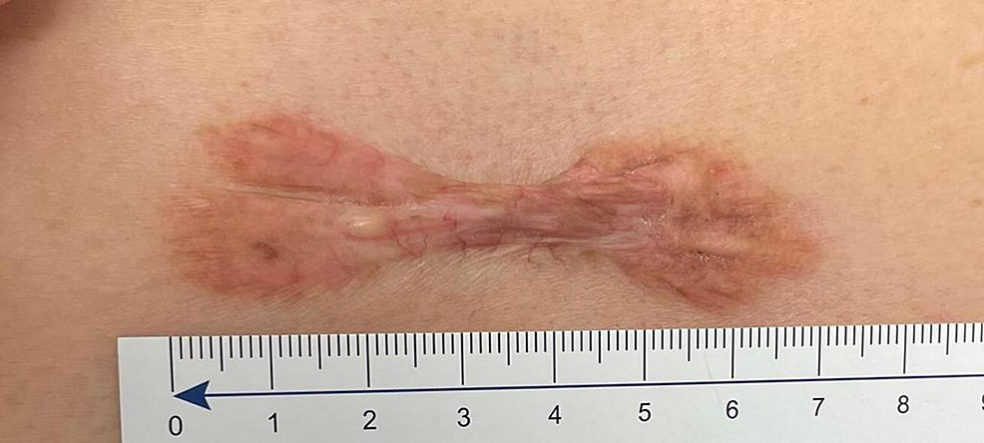
Keloid scar after six Kenalog injections

After the assessment, the patient was apprised of her treatment options which included steroid injections and surgical excision. The risks and benefits of each treatment option were discussed, such as the potential of further keloid formation, wound dehiscence with surgical excision, discoloration, and the possibility of requiring multiple treatments of steroid injections. The patient consented to the intralesional steroid injections that contained a mixture of 1 ml Kenalog-40 with 1 ml of lidocaine. The patient's treatment at the conclusion of this study consisted of six injections given over three to four-week intervals. Three months after initial presentation, the spontaneous keloid scar decreased to a size of 7 cm x 2 cm. The regression rate in the size of the spontaneous keloid scar is consistent with the latest measurement of 7 cm x 1.8 cm (Figure 2). The spontaneous keloid palpated to be softer to touch, lighter in color, and the patient reported relief of pruritus and paresthesia.

## Discussion

Although there have been several advancements made in providing treatment options, the majority of these options have proven to be insufficient in preventing the reappearance of keloids as well as adequately treating them. This case study presented evidence of improvement in the appearance and the symptoms associated with keloids in a patient that received six Kenalog-40 injections following a 10-year history of multiple treatment failures.

Keloids are more likely to be found in areas of the body that have high skin tension. From a histological perspective, a hypertrophic scar exhibits collagen in a wavy, parallel pattern while keloids display an abundance of collagen settled in a haphazard way or whorls [1]. As aforementioned, there is a higher incidence of keloids in people of Asian and African descent which positively correlates with increased melanin [4]. Spontaneous keloids are seen in those with novel X-linked disorders such as Dubowitz syndrome, Noonan syndrome, and Goeminne syndrome [3]. There is also a positive correlation between keloids and known human leukocyte antigens such as HLA-B14, HLA-B21, HLA-BW16, HLA-BW35, HLA-DR5, and HLA-DQW3 [7]. Interestingly, evidence suggests that people with blood group A have a higher genetic association with the development of abnormal scars in general [7-10]. 

The healing process has a balanced role between growth factors and cytokines. Several proinflammatory cytokines such as interleukin-1 (IL-1), interleukin-6 (IL-6) and tumour necrosis factor-alpha (TNF-α) as well as several growth factors, such as epidermal growth factor (EGF), fibroblast growth factor (FGF-2), transforming growth factor-beta (TGF-β), platelet-derived growth factor (PDGF), and vascular endothelial growth factor (VEGF) play intricate roles in achieving wound healing and eventual restoration [11]. Usually, interferon-alpha (IFN-α), interferon-beta (IFN-β), and interferon-gamma (IFN-ɣ) reduce fibroblast synthesis of type I, III, and VI collagen [7]. In keloid formation, there is an increased production of TNF-α, IFN-β, IL-6, and diminished IFN-a, IFN-ɣ, and tumour necrosis factor-beta (TNF-β) [7]. This dysfunction increases collagen production 20-times higher than a typical scar with an abundance of type III collagen, chondroitin 4-sulfate, and glycosaminoglycan, leading to an excessive formation and overgrowth at the site of injury [7]. 

There is currently no treatment for keloids that are considered the gold standard. Current therapeutic strategies are occlusive dressings, pressure therapy, corticosteroids, excisional surgery, radiation, cryosurgery, laser therapy, interferon therapy, skin tension offloading device, 5-fluorouracil, Imiquimod, flurandrenolide tape, bleomycin, tacrolimus, methotrexate, pentoxifylline, and colchicine [7]. Moreover, topical zinc, intralesional verapamil, cyclosporine, D-penicillamine, relaxin, and topical mitomycin C have shown limited success [7]. 

In the early 1980s, silicone-based products were implemented as a treatment option for keloids [12]. The assumption was that the silicone would enhance hydration and create an occlusive environment; thereby, improving the appearance of the scar tissue [12]. The silicone gel sheets have shown to be beneficial in reducing pain, tenderness, itching, and smoothening of the keloid while only causing minimal local irritation [12]. 

Pressure therapy has been used as an adjunct with surgery and intralesional steroid injections to decrease the appearance of keloid scars and reduce the risk of recurrence [13]. A study by Bran et al. showed that reduction in keloid scar size, pain and dysesthesia were evident when a pressure device was used after patients underwent surgery and intralesional steroid injections [13]. However, the authors focused on auricular keloids that developed after a known trauma to the area [13]. 

Surgical excision, either extra-lesional or intralesional excision, is one of the more conventional methods [14]. There is an increase in the likelihood of recurrence with extra-lesional excision versus intralesional excision [14]. Surgical excision is not recommended as a monotherapy and is incorporated with other therapies such as radiotherapy or corticosteroids [12]. Corticosteroids work to inhibit VEGF, TGF- β1, fibroblastic growth, reduction of endothelial budding, and enhancement of collagen and fibroblast degeneration [15]. 

A randomized parallel-group study compared the role of Kenalog, fractional CO2 laser, and intralesional verapamil in treating keloids in 60 patients [16]. The results showed a reduction in scar height, pliability, and vascularity in all groups and there was a statistically significant faster response when verapamil and laser therapy followed Kenalog injections [16]. 

Kenalog injections as a monotherapy are usually administered in intervals of four to six-week duration until the symptoms associated with the keloids have diminished [6,12]. Kenalog monotherapy has shown to have adequate efficacy and good clinical outcomes such as reduced keloid height, width, length, erythema, pruritus, and improved pliability [12]. However, there is up to a 50% recurrence rate with the use of Kenalog injections [1,6]. Although symptoms can be mild such as burning with injection, telangiectasia development, atrophy, and pigmentary changes [6,16] are aesthetically displeasing and a limiting aspect of repeated injections [6].

## Conclusions

Various medical and surgical treatment options for keloid scars are currently available on the market, demonstrating the difficulty in finding the most optimal management strategy. However, Kenalog injections are one of the most effective first-line treatments for keloid scars and it can be effective as a monotherapy for reducing the size of the scar as well as associated discomfort. Surgical excision as monotherapy has a high probability of forming new hypertrophic scars that may or may not worsen the original scar(s). 

Monotherapy with Kenalog injections has shown to be effective. Nevertheless, the addition of other treatment options could be considered depending on the severity, multiplicity, and location of the scars as well as the patient’s preference. For the patient in this case report, there was a reduction in size and overall appearance. However, the most important outcome was the improvement in pruritus and paresthesias. In conclusion, more research and initiatives must be explored to improve the appearance of keloids so that a more robust treatment option may be utilized in appeasing both symptoms and aesthetics. 
